# Strengths Against Psychopathology in Adolescents: Ratifying the Robust Buffer Role of Trait Emotional Intelligence

**DOI:** 10.3390/ijerph17030804

**Published:** 2020-01-28

**Authors:** José A. Piqueras, Maria do Céu Salvador, Victoria Soto-Sanz, Francisco Mira, Juan-Carlos Pérez-González

**Affiliations:** 1 Department of Health Psychology, Faculty of Social and Health Sciences, Campus of Elche, Miguel Hernandez University (UMH), Elche 03202, Spain; jpiqueras@umh.es (J.A.P.); paco.mira14@gmail.com (F.M.); 2 Center for Research in Neuropsychology and Cognitive and Behavioral Intervention, University of Coimbra, Coimbra 3001-115, Portugal; ceusalvador@gmail.com; 3 Emotional Education Laboratory (EDUEMO Lab), National University of Distance Education (UNED), 28040 Madrid, Spain

**Keywords:** youth mental health, trait emotional intelligence, mindfulness, catastrophizing, cross-cultural research

## Abstract

The aim of this study was to unravel the interrelated effects of trait emotional intelligence (Trait EI), mindfulness, and irrational beliefs on adolescent mental health. A random sample of students from three secondary schools in Spain and eight secondary schools in Portugal was recruited. We conducted four-step hierarchical regression analyses. We also conducted regression analyses to examine the role of mindfulness skills and catastrophizing as mediators of the link between emotional intelligence and psychosocial problems. Finally, the SPSS PROCESS computing tool was used to perform conditional process analysis (model 6). A total of 1370 adolescents from Spain (*n* = 591) and Portugal (*n* = 779) participated in this study (mean age = 14.97, SD = 1.50; range = 12–18). The mediation analyses confirmed that adolescent mental health was determined by Trait EI directly, and by mindfulness skills and catastrophizing thoughts in an indirect way. Together, the four variables explained 44% of psychopathology, with EI being the most powerful predictor, which ratify the robust buffer role and incremental validity of Trait EI against youth mental health. The identified pathways provide keys for emotional education interventions aimed at promoting adolescent mental health.

## 1. Introduction

### 1.1. Mental Health in Adolescence

According to epidemiological studies, the worldwide-pooled prevalence of mental disorders in children and adolescents is around 13.4%, so it can be asserted that mental disorders affect a significant number of youths worldwide [1]. In addition, a large number of studies using symptom scales such as the Child Behavior Checklist (CBCL) and the Strengths and Difficulties Questionnaire (SDQ) to assess emotional and behavioral symptomatology or dimensional psychopathology have showed prevalence rates between 5% and 20%, with the mean rate being around 15% [2,3]. 

Considering the prevalence, assessment of child and adolescents mental health problems is of great importance, not only for public health, but also for clinical practice and research [4], given that numerous studies have linked the presence of problems in childhood with later psychopathological development in adulthood [5].

Among the determinants of adolescent mental health, previous studies have examined the role of protective factors or strengths such as emotional intelligence (EI) and mindfulness, and the role of risk factors, such as irrational beliefs (specifically catastrophizing). 

### 1.2. Trait Emotional Intelligence and Mental Health

Salovey and Mayor defined EI in 1990 [6] as the ability to control one’s own feelings and emotions, as well as to control the feelings and emotions of others and have positive control over social performance. In 2001, Petrides and Furham [7] created the trait model and conceptualized the term EI as a personality trait with 15 dimensions, 2 independent (Adaptability and Self-motivation) and the other 13 grouped according to 4 factors: Wellbeing, Self-control, Emotionality, and Sociability.

Martins, Ramalho, and Morin [8] conducted a large meta-analysis based on 105 effect sizes and 19,815 adult and adolescent participants (11 years and above), establishing that EI was a positive predictor of mental health. Extensive available evidence suggests EI is a buffer of impact of stressful circumstances on mental health, both in adolescents and in adults [9,10].

Keefer, Saklofske, and Parker [11] explain different pathways from EI to mental health, such as that: (i) High EI is associated with healthier emotional and physiological stress response; (ii) high EI is related with less use of avoidance, ignoring or distraction strategies to cope with stressful situations, and further associated with coping strategies such as dealing with the stressor directly; (iii) high EI is related with less use of passively worrying focused on a stressful situation or of numbing their feelings with substances, which could aggravate health risks.; and (iv) high EI is correlated with more adaptative ways of coping with health threats and chronic illnesses. Nonetheless, although the connection between EI and health has already been well established for adults, it has scarcely been replicated in children and adolescents to date.

This study is based on one of the most scientifically supported models currently available in both educational and clinical settings, the Trait EI theory formulated by Petrides and Furnham [7] and revised by Petrides et al. [12]. According to this EI model, Trait EI is a collection of affective self-perceptions (i.e., trait emotional self-efficacy) and dispositions which facilitate daily emotional competence [13,14]. Trait EI is considered to provide a more comprehensive operationalization of the affective aspects of personality than the general models of Big Five [15]. In summary, Trait EI can be understood as a personality trait that apprehends individual differences in recognition, processing, and regulation of emotionally charged information, with generally adaptive effects for social efficiency and emotional wellbeing [12,15,16].

A systematic review by Resurrección, Salguero, and Ruiz-Aranda [17] found that EI was negatively associated with adolescents’ internalizing problems, eating disorder symptoms, addictions, and maladaptive coping and positively associated with coping strategies. Subsequent studies using adolescent samples have generally found a direct negative effect of Trait EI on emotional and behavioral difficulties, an indirect effect through social skills [18], somatic complaints and self-report psychopathology, and a positive relationship with personal strengths [19].

### 1.3. Mindfulness and Psychopathology

Shapiro and Carlson [20] define mindfulness as “the awareness that arises through intentionally attending in an open, caring, and discerning way”. This mindful awareness involves experiencing present life events and inner experiences as they come and go in each moment. Several studies indicate that mindfulness correlates negatively with psychopathology, confirming its predictive capacity as a protective variable [9,21,22]. Likewise, according to Keye and Pidgeon [23], full attention explains resilience and is related to less distress in the face of childhood problems [24]. Therefore, mindfulness is a vehicle for emotional wellbeing, correlating with a range of positive and negative mental health variables in adolescents [25,26,27].

### 1.4. Irrational Beliefs: Catastrophizing

According to Ellis, David, and Lynn [28], irrational beliefs are unrealistic thoughts about oneself, the world, and life, playing a central role in emotional disturbance or psychopathology. The original 11 irrational belief types of Rational Emotive Behavior Therapy formulated by Ellis in 1962 [29] were later assigned to four main categories: demandingness (i.e., absolutistic/inflexible requirements), awfulizing (or catastrophizing), frustration intolerance (or low frustration tolerance), and global evaluation of one’s own person (self-downing), other persons (other-downing), and/or the life situation (life-downing) [28]. 

Specifically, catastrophizing can be defined as thoughts emphasizing the terror of what was experienced [30]. Psychological distress has been consistently and positively associated to catastrophizing, among other coping strategies [30,31,32]. 

A recent meta-analysis, including 100 independent samples, gathered in 83 primary studies with adult samples, corroborated a moderate (overall r = 0.38) but robust relationship between any type of irrational beliefs and various types of psychological distress, such as general distress, anxiety, depression, anger or guilt [32]. 

### 1.5. Relationships between Emotional Intelligence, Mindfulness, and Catastrophizing

Mindfulness promotes the development of emotional regulation and increases the recognition of one’s own and others’ emotions. Additionally, the nonjudgmental and self-regulating aspects of mindfulness may enable individuals to more accurately decode their own and others’ emotions and to possess better emotion management skills [33,34]. Mindfulness is associated with adaptive emotional functioning and helps to redirect individuals’ attention away from maladaptive processes, thereby minimizing psychological distress [33]. Hence, some of the essential aspects of trait mindfulness are related to emotion regulation and perception of emotions, which are also core components of EI. In fact, a recent meta-analytic review with adolescent and adult samples concluded that emotional intelligence was significantly associated with trait mindfulness (effect size = 0.49) [35].

A recent study carried out with a clinical sample of adults investigated possible pathways into mental illness via the combined effects of Trait EI, mindfulness, and irrational beliefs. These authors indicated that Trait EI constituted a stronger predictor of psychopathology than mindfulness and that mindfulness was a stronger predictor than irrational thoughts. The study points out that Trait EI has direct effects on psychopathology and indirect effects through mindfulness and that Trait EI is a stronger predictor of psychopathology than mindfulness and irrational beliefs combined [10]. 

### 1.6. Cross-Cultural Differences on Studies Variables

According to Polanczyk et al. [1], a large number of studies in a wide variety of countries assessing child and adolescent psychopathology from a dimensional point of view have found more similarities than differences in terms of psychopathology, showing association between them and only slight differences in symptom rates [2,3,36]. However, since cultural diversity cannot be dissociated from variation in study methods in the previous literature, no conclusions can be drawn about the independent effect of culture and social aspects over prevalence estimates.

Concerning Trait EI, some studies have examined cross-cultural differences in adolescent Trait EI, focusing specifically on comparing individuals from individualistic versus collectivist societies. For example, Gökçen, Furnham, Mavroveli, and Petrides [37] found that Chinese participants who completed the English version of the TEIQue scored higher on global Trait EI and the TEIQue factor of sociability compared to Chinese participants completing the TEIQue in their native language. Nozaki [38] compared European-American and Japanese populations, showing a moderator effect of culture on emotion regulation process that underlies the Trait EI.

Regarding irrational beliefs cross-culturally, studies have focused on investigating cross-cultural differences in the use of cognitive strategies and on testing whether the relationship between specific strategies and psychopathology varies across countries. A recent study [39] found that there were differences in strategies that have been associated with symptoms of psychopathology; overall, Northern European countries (Germany and the Netherlands) had less use of rumination, catastrophizing, and other-blame compared to Southern and Eastern European countries (Spain, Italy, Portugal, and Hungary) but not among southern countries.

Finally, there are very few cross-cultural studies on trait mindfulness [40,41], indicating absent or small to absent cross-cultural differences in trait mindfulness between different countries and languages. 

There is only one study that has explored the combined effects of Trait EI, mindfulness, and irrational beliefs on mental disorders in a clinical sample of adults [10], indicating that Trait EI, mindfulness, and irrational beliefs constituted powerful predictors of psychopathology, with a stronger effect of Trait EI by means of its direct effect and indirect effects through mindfulness and irrational beliefs.

Despite this, little consolidated research has been carried out on the combined effects of Trait EI, mindfulness, and catastrophizing thoughts on psychopathology specifically in populations of adolescents. Particularly, there is little available cross-cultural research that has explored the mediating effect of country of origin in the relationships between Trait EI and adolescent psychopathology.

Consequently, this study aims to provide evidence of the direct influence of the Trait EI on adolescent psychopathology and its indirect (mediational) influence through indicators of mindfulness and irrational beliefs (catastrophizing). In addition, this study examines whether the country of origin has a moderating effect on the relationship between Trait EI and the level of psychopathology in a sample of Spanish and Portuguese adolescents.

Based on our previous review of the literature and according to our rationale, the following hypotheses have been put forward: Psychopathology is negatively associated with Trait EI and mindfulness and positively associated with catastrophizing (Hypothesis 1); Trait EI, mindfulness, and catastrophizing are significant predictors of psychopathology (Hypothesis 2); Trait EI predicts psychopathology through its direct effect and through an indirect effect through mindfulness and catastrophizing (Hypothesis 3); this prediction exists above any effect of the country of origin (Hypothesis 4). This research was exploratory, and the hypotheses were not preregistered.

## 2. Materials and Methods

### 2.1. Participants

Using the nonprobability and incidental sampling method, a random sample of students from 3 secondary schools in the city of Elche (Spain) and from 8 secondary schools in the central region of Portugal was recruited. Of the total population recruited, data on 129 adolescents were eliminated because the questionnaires were not entirely completed, so a final sample of 1370 participants was established: 591 Spanish adolescents (43.1%) and 779 Portuguese (56.9%). The sample included students from the 7th to the 12th year of school. Statistically significant interdependence was found between the country and the courses, with a significant Chi-square value (χ² = 70.55, *p* < 0.001), although the size of the association’s effect size was small (Cramer’s V = 0.23).

In both Spanish and Portuguese samples, the distribution of boys and girls was similar. Thus, there were 299 boys (50.6%) and 292 girls (49.4%) in the Spanish sample and 348 boys (44.7%) and 431 girls (55.3%) in the Portuguese sample, with a value of χ² =.4.73 and *p* = 0.03. However, the effect size was again small (Cramer’s V = 0.06). In terms of age, the participants ranged between the ages of 12 and 18. The average age of the samples was 14.97 (SD= 1.50) for the Spanish sample and 14.64 (SD = 1.47) for the Portuguese sample. In this case, *t*-Student was performed and a value of *t* = 4.09 (*p* < 0.001) was obtained, but with a small effect size (*d* = 0.22).

The exclusion criteria were (a) refusal to participate in the study, (b) students not enrolled in secondary education, (c) under age 12, and (d) over age 18.

### 2.2. Variables and Instruments

The alpha coefficients for all the variables studied are shown in Table 1.

Information on sociodemographic variables (sex, age and course) was obtained through an ad hoc questionnaire. 

The *Strengths and Difficulties Questionnaire* (SDQ) [42] was used to assess emotional and behavioral symptoms. The Portuguese and Spanish self-reported versions for adolescents were applied (they are available from: http://www.sdqinfo.org/py/sdqinfo/b0.py). The questionnaire is made up of 25 items with a Likert-type scale of three response options. It has five subscales consisting of 5 items each: emotional symptoms, behavior problems, peer problems, hyperactivity, and prosocial behavior. The sum of the first four constitutes the total difficulties score. The psychometric properties of the SDQ have been examined in previous studies both for Spanish [36,43] and for Portuguese adolescents [44,45].

The *Trait Emotional Intelligence Questionnaire-Adolescent Short Form* (TEIQue-ASF) [46] was used to evaluate trait-emotional intelligence (Trait-EI), specifically, the Adolescent Short Form available in Spanish and in Portuguese (retrieved from http://www.psychometriclab.com). The ASF form was designed to improve the understanding of the items, taking into account syntax and formulation elements, resulting in a simplified version of the adult abbreviated form of the TEIQue. It consists of 30 items, two for each of the 15 aspects of Trait EI. The sum of all items indicates the total score of Trait-EI. This instrument has shown adequate psychometric properties for Spanish [47] and Portuguese adolescents [48].

To evaluate mindfulness, The Children’s Acceptance and Mindfulness Measure (CAMM) [49] was used, adapted to Spanish by the research group on Childhood, Adolescence, Children’s Rights and Quality of Life (ERIDIQV) of the University of Girona (http://www.udg.edu/eridiqv) and to Portuguese by Cunha, Pinto-Gouveia, and Paiva [50]. It is a self-administered scale composed of 10 items to assess the degree of full attention in children and adolescents. It presents a unifactorial structure and a Likert-type format with 5 response options. The CAMM items measure three aspects of mindfulness: awareness of internal phenomena; acting with awareness in the present moment; and experimentation without making critical judgments. The CAMM has shown good psychometrics for Portuguese [51,52], Catalan-speaking Spanish [53], and Spanish-speaking Spanish adolescents [54].

The catastrophizing subscale of the *Cognitive Emotion Regulation Questionnaire* (CERQ) was used [30]. The CERQ was designed to measure the emotional regulation strategies used in the face of threats or stressful life events. It is composed of 36 items that give rise to nine subscales: self-blame; acceptance; rumination; positive reorientation; planning; positive reappraisal; putting into perspective; catastrophizing; and other-blame. Each subscale has four items that are evaluated using a Likert-type scale with five response options. For this study, we only used the catastrophizing subscale. The Spanish version and the Portuguese version of the CERQ were used [55,56]. Both versions have shown good psychometrics for Spanish [57] and Portuguese samples of adolescents [58,59]. 

### 2.3. Procedure

This was a cross-sectional, observational, and cross-cultural study of adolescents in secondary schools in the province of Alicante (Spain) and the central region of Portugal. 

To carry out this work, prior approval was obtained from the Ethics Committee of the Miguel Hernández University (project identification code: DPS.JPR.02-17) and the National Data Protection Commission (CNPD), and the Ethics Committee of the Faculty of Psychology and Educational Sciences of the University of Coimbra and also from the Directorate General for Curriculum Innovation and Development (DGIDC) in Portugal. With regard to student participation, the schools were responsible for obtaining the relevant informed consent.

The data collection took place within the context of the classroom during a unique protocol administration in the academic year 2017/2018. The participation of the students was voluntary and anonymous, and they could stop participating at any time. The assessments were administered in pencil and paper. Students in the final year of their psychology degree carried out the data collection. The data are not made openly accessible because the consents did not explicitly request agreement to share such data, although they were unlinked data and there would be no way to identify the participants.

### 2.4. Data Analyses

**Figure 1:**
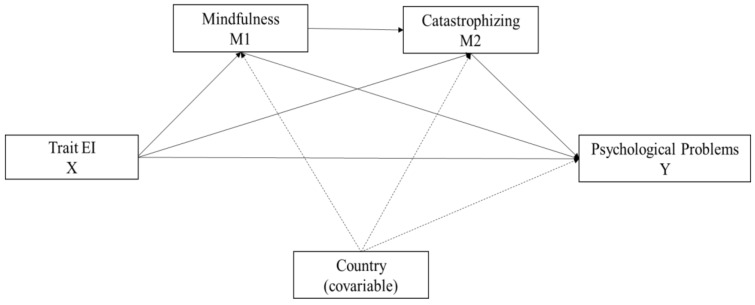
Conceptual and statistical diagram of Model 6 with 2 mediators plus a covariate.

First, preliminary analysis was carried out on the descriptive statistics. Consequently, we compared mean scores on variables by country, providing effect sizes (Cohen’s d). In addition, we calculated internal consistencies for measures, and we compared alpha coefficients by means of Package “Cocron” [60]. Secondly, analyses of the relationship between emotional intelligence, mindfulness skills, catastrophizing, and risk for psychological problems were carried out using Pearson’s correlation coefficient, following Cohen’s [61,62] recommendations to consider a correlation as indicative of a small (less than 0.29), medium (between 0.30 and 0.49) or large (0.50 or greater) effect size. Finally, the SPSS PROCESS computing tool [63,64] was used to perform conditional process analysis (model 6). Therefore, as shown in Figure 1, unstandardized regression coefficients were estimated using the bootstrapping procedure (10,000 resamples) that results in a 95% corrected bias and direct and indirect effect confidence intervals, where they are considered significant if zero is not present between the upper and lower confidence intervals (CI). It was considered significant when the value was less than 0.05.

## 3. Results

### 3.1. Mean Scores, Internal Consistencies, and Bivariate Correlations

As can be seen in Table 1, there were statistically significant but small differences between estimates of internal consistency by country for the CAMM score (*p* < 0.001) and for the CERQ-Catastrophizing subscale (*p* < 0.05).

Concerning comparison in variables by country, the mean score on CAMM and TeiQue-ASF was statistically higher in Spanish adolescents than in Portuguese adolescents, with a medium effect size. Further, Portuguese adolescents had higher SDQ scores, but the magnitude of the difference was small (see Table 2). 

The correlations between all variables are shown in Table 3.

### 3.2. Mediation Anaylisis

**Figure 2:**
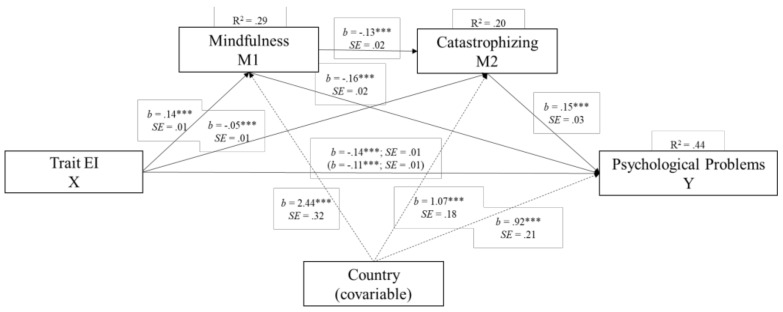
Double mediation model of the relationships among trait emotional intelligence, mindfulness skills, catastrophizing thoughts, and psychological problems, after controlling for country of origin (Portugal vs. Spain). Statistical diagram of the double mediation model for the expected influence of EI and its association through mindfulness and catastrophizing on psychological problems, and the country as a covariate. The values are represented with unstandardized regression coefficients. In the association between Trait EI and psychological problems, the value outside brackets represents the total effect, and the value in brackets represents the direct effect of the bootstrapping analysis of Trait EI on Psychological Problems after the inclusion of the mediating variables. *** *p* < 0.001.

Preliminary analysis of regression showed that Trait EI, mindfulness skills, and catastrophizing were individual predictors of psychological problems and that when they were introduced simultaneously in the same model (controlling for the country of origin), they accounted for 44% of the variance in psychosocial adjustment. These results suggest that all the constructs are relevant in determining adolescents’ psychological problems. To further explore this idea, we built a mediation model to test the relationship between these variables, and we investigated the hypothesis that mindfulness skills and catastrophizing would be important mechanisms in the relationship between Trait EI and the risk of psychological problems in adolescents (see Figure 2). The country of origin was included as a covariate.

As can be seen in Route C, Figure 2, this mediation analysis showed that adolescents with lower Trait EI were at a higher risk of psychological problems. Furthermore, the indirect effect was significant (c’ = −0.03, Standard Error [SE] = 0.003). In addition, Route A in Figure 2 shows that Trait EI was positively associated with mindfulness skills and negatively associated with catastrophizing, suggesting that adolescents with a higher level of Trait EI have higher mindfulness skills and fewer catastrophizing thoughts. Finally, as can be seen in Route B in Figure 2, mindfulness skills were negatively related to psychological problems, while catastrophizing was positively related to the risk of psychological problems.

Regarding the influence of the country of origin, this variable was significantly associated with mindfulness, catastrophizing, and psychological problems: Being a Portuguese adolescent meant being more prone to having fewer mindfulness skills, more catastrophic thoughts, and more psychological problems. Nevertheless, as was seen before, all the other variables also had a significant effect on the dependent variable.

## 4. Discussion

The aim of this study was to examine how Trait EI, mindfulness, and catastrophizing thoughts affected the dimensional psychopathology of adolescents. As a further step, this study examined whether mindfulness and catastrophizing played a role in mediating the relationship between Trait EI and psychopathology, controlling for the country of origin.

Globally, mediation analysis revealed the multiple and complex pathway in which psychopathology in adolescents was determined by Trait EI, mindfulness, and catastrophizing. 

In particular, concerning the first hypothesis, there was a negative association between mindfulness and dimensional psychopathology. This result is similar to results obtained in previous research, asserting that mindfulness is a vehicle for emotional wellbeing, correlating with a range of positively and negatively mental health variables in adolescents [22,25,26,27]. Further, lower scores in Trait EI seemed to predict worse mental health (higher scores in the SDQ). This result is consistent with extensive previous evidence, revealing that high Trait EI is a positive predictor of mental health [8,10,17,19,65]. On the other hand, catastrophizing had a positive relationship with the total score of SDQ, that is, the more the adolescents think in catastrophic ways, the more the difficulties during adolescence. This finding is consistent with previous literature pointing out that awfulizing or catastrophizing are a type of irrational belief related with psychological distress and dimensional psychopathology [30,31,32].

Trait EI, mindfulness, and catastrophizing significantly predicted psychopathology, therefore confirming Hypothesis 2. In line with Hypothesis 3, Trait EI exerted a negative direct effect on psychopathology, as well as an indirect effect, through mindfulness skills and catastrophizing. This result suggests that mindfulness skills and catastrophizing partially mediate the association between Trait EI and psychological problems. The results obtained in this study are similar to those found in previous studies with adults and represent an advancement in scientific knowledge in the comprehension of adolescents’ psychopathology. Thus, this study was intended to replicate the study of Petrides et al. [10], and it also pointed out that emotional intelligence, reasoning processes and maintaining attention to the present moment play an important role in the expression and maintenance of mental disorders. In other words, Trait EI, mindfulness skills, and catastrophizing seem to explain much of the adolescent difficulties. In addition, we found the same percentage of explained variance as Petrides et al. [10] in adults (44%). Furthermore, our findings were also consistent with recent studies including Trait EI as the main predictor of different measures of psychosocial adjustment. Accordingly, Piqueras, Mateu, Cejudo, and Perez-Gonzalez [66] found that psychosocial adjustment in children was determined by Trait EI directly and by emotional and social problems indirectly. Together, the three variables explained 46% of the variance in psychosocial adjustment, although Trait EI was the most powerful predictor (44%). Likewise, Petrides et al. [10] found that the direct effect of Trait EI on psychopathology was the higher effect in a clinical sample of adults. Therefore, mindfulness and Trait EI constitute a vehicle for emotional wellbeing by positively influencing variables such as resilience and adaptive coping styles [10,14,23,25]. Meanwhile, catastrophizing constitutes a risk factor in the expression and maintenance of mental disorders [10,32].

Regarding the fourth hypothesis, although there were differences between Portuguese and Spanish adolescents in the variables under study, the model explained dimensional psychopathology over and above the effect of the country of origin. In fact, we found that mindfulness skills, catastrophic thoughts, and EI were influenced by cross-cultural differences, with adolescents from Spain reporting higher scores for mindfulness skills and for EI. This finding would show the role of the country of origin as a possible facilitator of the link between mindfulness skills, catastrophizing, and psychological problems. Although it was an interesting result, we could not compare it with other studies due to the scarce cross-cultural studies exploring differences between close cultures, countries or territories. Nevertheless, this result may mean that Portuguese adolescents, compared to Spanish adolescents, may be more prone to developing psychopathology due to having less EI and mindfulness and using more catastrophizing.

In terms of limitations, a transversal design has been used, preventing the demonstration of the temporary origin necessary to establish causal inferences. On the other hand, all measures were self-reported. Along these lines, in relation to the development of certain research gaps, future studies should use a longitudinal design, a bigger sample, and make use of probability sampling techniques. Moreover, future studies are needed to replicate these findings and to further explore the effects of country of origin on Trait EI, mindfulness, irrational beliefs, and psychopathology.

Finally, despite these limitations, results confirmed the hypothesis initially proposed and provide a new vision for understanding the effects of Trait EI, mindfulness, and catastrophizing on adolescents’ psychosocial adjustment. Specifically, this study ratifies the robust buffer role and the incremental validity of Trait EI in the prediction of youth psychopathology. 

## 5. Conclusions

These findings replicate in adolescents the positive association between emotional intelligence and mental health previously observed in young people and adults [8,10,12]. In particular, this study empirically supports the interpretation of Trait EI as a protective factor for the development of psychopathology in adolescents. 

One of the core contributions of this study was its evolutionary perspective, focused on adolescents, since most of the research published so far has been carried out on adults. Research carried out on child and adolescent emotional intelligence and psychopathology is still incipient [67], despite the personal and psychosocial consequences of difficulties in diverse areas (such as emotional, cognitive and social) or the problems of imbalance at this developmental period [68,69,70]. 

From an applied viewpoint, the enhancement of knowledge on the factors that influence Trait EI can aid in developing tools and interventions to promote mental health and to help adolescents to adapt during one of the most conflictive periods of developmental transition [71]. It looks promising to design emotional education programs to promote adolescent Trait EI, including those variables that research has identified as the most relevant ones, in order to prevent problems of maladjustment in adolescence. 

In summary, the results of this study highlight the pertinence of integrating components of traditional cognitive-behavioral therapies (such as Ellis’ rational-emotive behavioral theory) with the new generation of behavioral therapies, such as mindfulness-based and emotional intelligence models.

## Figures and Tables

**Table 1:** Estimates of reliability and comparison of Cronbach’s alpha coefficients.

Variable	Spain	Portugal	Total Sample	Cronbach’s Alpha	Number of Items
SDQ	0.67	0.63	0.64	0.16	25
TeiQue-ASF	0.85	0.85	0.86	0.56	30
CAMM	0.85	0.76	0.82	0.0001	10
CERQ-Catastrophizing	0.73	0.78	0.75	0.04	4

*Note.* SDQ: total score on the Strengths and Difficulties Questionnaire; TeiQue-ASF: total score on Trait Emotional Intelligence Questionnaire-Adolescent Short Form; CAMM: total score on Children´s Acceptance and Mindfulness Measure; CERQ-Catastrophizing: Catastrophizing subscale of the Cognitive Emotion Regulation Questionnaire.

**Table 2:** Variable mean scores by country.

Variable	Spain	Portugal	*t* (1368)	*d*
SDQ	11.44 (5.20)	12.15 (4.74)	−2.61 **	−0.14
TeiQue-ASF	146.06 (22.65)	136.42 (21.09)	8.12 ***	0.44
CAMM	25.69 (7.24)	21.88 (5.77)	10.85 ***	0.59
CERQ-Catastrophizing	9.39 (3.71)	9.28 (3.55)	0.57 ns	-

*Note*. SDQ: total score on the Strengths and Difficulties Questionnaire; TeiQue-ASF: total score on Trait Emotional Intelligence Questionnaire-Adolescent Short Form; CAMM: total score on Children´s Acceptance and Mindfulness Measure; CERQ-Catastrophizing: Catastrophizing subscale of the Cognitive Emotion Regulation Questionnaire. ** *p* < 0.01. *** *p* < 0.001.

**Table 3:** Descriptive statistics and bivariate correlations among the measures.

Variables	Mean	SD	1	2	3	4
(1) Trait EI	140.58	22.29	-			
(2) Mindfulness skills	23.52	6.72	0.51 **	-		
(3) Catastrophizing	9.32	3.62	−0.38 **	−0.35 **	-	
(4) Psychological problems	11.84	4.96	−0.62 **	−0.48 **	0.38 **	-

*Note*. Trait EI: Trait Emotional Intelligence. ** *p* < 0.01.
